# A Multicenter Study of the Distribution Pattern of Posterior-To-Anterior Corneal Curvature Radii Ratio in Chinese Myopic Patients

**DOI:** 10.3389/fmed.2021.724674

**Published:** 2021-12-20

**Authors:** Changting Tang, Qiaowei Wu, Baoyi Liu, Guanrong Wu, Jing Fan, Yijun Hu, Honghua Yu

**Affiliations:** ^1^The Second School of Clinical Medicine, Southern Medical University, Guangzhou, China; ^2^Department of Ophthalmology, Guangdong Eye Institute, Guangdong Provincial People's Hospital, Guangdong Academy of Medical Sciences, Guangzhou, China; ^3^Affiliated Hospital of Guilin Medical University, Guilin, China; ^4^Refractive Surgery Center, Aier Institute of Refractive Surgery, Guangzhou Aier Eye Hospital, Guangzhou, China; ^5^Aier School of Ophthalmology, Central South University, Changsha, China

**Keywords:** P/A ratio, corneal refractive power, refractive and cataract surgery, myopia, IOL power

## Abstract

Estimation of corneal refractive power (CRP) is of crucial importance to refractive and cataract surgery. The ratio of posterior to anterior curvature radii of the cornea (P/A ratio) is one of the key factors to determine the actual CRP (True-K). While the traditional method to calculate the CRP (Sim-K) is based on a constant P/A ratio (0.82), it is suggested that the P/A ratio varies in different people and exhibits a distribution pattern, which may have an impact on the accuracy of CRP estimation and postoperative refractive outcome. In this multicenter study, we aimed to investigate the distribution pattern of the P/A ratio in a large number of myopic patients, and further explore the relationship between P/A ratio and ΔK (the difference between True-K and Sim-K). We found that distribution of the P/A ratio ranged from 0.72 to 0.86 with an average value of 0.82 ± 0.01. The compensation effect of the refractive power of the posterior on the anterior surface of the cornea decreased with the increase of P/A ratio. There was a significant correlation between P/A ratio and ΔK in all eyes (*r* = 0.9764, *P* < 0.0001). A change of 0.1 in P/A ratio could cause a change of 0.75 D in ΔK. Our study suggests that the actual P/A ratio should be taken into consideration in refractive and cataract surgery when calculating the CRP and power of the intraocular lens in eyes with significantly deviated P/A ratios.

## Introduction

The corneal refractive power (CRP) accounts for 2/3 of the total refractive power of human eyes and it is essential in refractive and cataract surgery. A small change in the CRP can lead to a significantly alteration of the refractive state. Accurate estimation of the CRP is important for achieving satisfactory postoperative visual acuity after refractive and cataract surgery. According to recent studies, errors in calculation of the intraocular lens (IOL) power mainly stem from inaccurate measurement of the axial length, anterior chamber depth and CRP (1, 2). In another study, 3.69% of the refractive errors after cataract surgery are caused by the wrong P/A ratio (3).

In the past, keratometers and corneal topographers could only analyze the anterior surface of the cornea (4). Therefore, the traditional method used to calculate CRP is based on measurement of the anterior cornea, ignoring the importance of the posterior corneal surface. In this method, using a fixed P/A ratio of 0.82, a corneal thickness of 500 μm, and a corneal refractive index of 1.3375, the simulated corneal curvature (Sim-K) is calculated based on the radius of curvature of the anterior corneal surface. Currently, the anterior segment imaging system such as Pentacam and Galilei can simultaneously obtain information on the anterior and posterior surfaces of the cornea as well as the thickness of cornea, making it possible to analyze the P/A ratio distribution pattern among people and also the actual CRP (True-K) which is directly derived from the anterior and posterior corneal radii of curvature (5, 6).

Previous studies have shown that there are some discrepancies between the Sim-K and True-K. A difference of about 0.6 D is found when comparing the True-K obtained by Pentacam and Sim-K provided by keratometer (7–9). Similarly, the True-K measured by Galilei is about 0.4 D lower than the keratometric Sim-K (10, 11). True-K has been proved to improve the accuracy of intraocular lens power calculation (12). Especially, in eyes with previous corneal refractive surgery and posterior keratoconus, the thickness and curvature of cornea has been changed and the True-K is much more accurate to the IOL power calculation than the Sim-K (13–16). In addition, the difference between True-K and Sim-K (ΔK) could be significantly dependent on the P/A ratio. Thus, a systematical investigation based on a large dataset can shed light on the P/A ratio distribution pattern and its relationship with ΔK.

So far there is little information of the P/A ratio distribution pattern in Chinese myopic adults, which is the largest group of refractive surgery candidates in the world. In this study, we collected data from five ophthalmic centers to investigate the distribution pattern of corneal P/A ratio and reveal the relationship between P/A ratio and ΔK in Chinese myopic patients. Our results could be of clinical significance and implications in myopic refractive and cataract surgery.

## Methods

### Participants

This retrospective study conformed to the tenets of the Declaration of Helsinki and was approved by the Institutional Review Board (IRB) of Guangzhou Aier Eye Hospital (GZ), Shenyang Aier Eye Hospital (SY), Wuhan Aier Eye Hospital (WH), Chengdu Aier Eye Hospital (CD) and Hankou Aier Eye Hospital (HK). It was only a review of medical records and patients could not be identified from the data, so the IRBs decided to waive the need to obtain informed consent (17, 18). We reviewed the digital medical records of myopic patients who underwent refractive surgery from 2017 to 2020 in the five ophthalmic centers, and eyes meeting inclusion criteria were included consecutively. Inclusion criteria were myopic patients with a spherical equivalent (SE) ≤ −0.50 D and Scheimpflug scans of good quality. In the present analysis, we included the right eye of each patient only. Exclusion criteria were coexisting corneal diseases, keratoconus (such as a significantly asymmetrical bowtie, a posterior elevation value of ≥+15 at the thinnest point with red spot on Belin/Ambrosio Enhanced Ectasia Display) (19), forme fruste keratoconus (such as the follow eye of patients with unilateral keratoconus) (20), severe dry eye, previous ocular trauma or surgery, uveitis, glaucoma, wearing contact lenses within the previous 2 weeks, age younger than 18 years (unstable refraction) or older than 40 years (to reduce the effects of the crystal lens on refraction) (17, 18).

### Examinations

All the patients underwent routine preoperative examinations including best-corrected visual acuity (BCVA), intraocular pressure (IOP), cycloplegia and manifest refraction, slit lamp examination of anterior segment, corneal topography and Pentacam measurements. The eyes were divided into four myopia groups according to the manifest SE: low myopia (LM, −3.00 D < SE ≤ −0.50 D), moderate myopia (MM, −6.00 D < SE ≤ −3.00 D) and high myopia (HM, −10.00 D < SE ≤ −6.00 D) and ultra-high myopia (UHM, SE ≤ −10.00 D), or four astigmatism groups according to the manifest astigmatism (MA): slight astigmatism (SMA, MA <0.50 D), low astigmatism (LMA, 0.50 D ≤ MA <1.00 D), moderate astigmatism (MMA, 1.00 D ≤ MA <2.00 D), high astigmatism (HMA, MA ≥ 2.00 D).

Pentacam examinations were performed for the patients by experienced technicians. Pentacam has high reliability in the measurement of corneal parameters, with a repeatability of anterior and posterior corneal curvature of ±0.28 D and ±0.11 D, respectively (21). It uses rotating Scheimpflug imaging technology to collect digital images of the entire anterior segment including the morphology of the anterior and posterior surfaces of the cornea (22). The Pentacam instrument (Pentacam HR, Oculus GmbH, Wetzlar, Germany) was calibrated regularly on a weekly basis. Details and quality control of Pentacam examination were described previously (17, 18).

### Data Analysis

Sim-K is the simulated corneal curvature calculated by using the standardized corneal refractive index (1.3375) and the radius of curvature of the anterior surface of the cornea. True-K is the equivalent corneal refractive power which is calculated based on Gaussian optics formula. ΔK is the difference between True-K and Sim-K (True-K minus Sim-K). Formulas to obtain the CRP are shown as follows:


(1) $$\begin{array}{l} Sim\text{-}K=\;(1.3375-n_{0})/R_{anterior}\\ True\text{-}K=(n_{1}-n_{0})/R_{anterior} \end{array}$$

(2) $$\begin{array}{l} +(n_{2}-n_{1})/R_{posterior}-(CCT/n_{1})\times \left[(n_{1}-n_{0})/R_{anterior}\right]\\ \times \left[(n_{2}-n_{1})/R_{posterior}\right] \end{array}$$

where *n*_0_ is the refractive index of air (=1), *n*_1_ is the refractive index of the cornea (=1.376) and *n*_2_ is the refractive index of the aqueous humor (=1.336), CCT is the central corneal thickness, *R*_anterior_ and *R*_posterior_ are the mean anterior and posterior corneal curvature radii, respectively (1, 9, 11).

All analyses were performed using InStat (GraphPad Software, version 8.0.2). Kolmogorov-Smirnov (KS) test was used to evaluate the normality of all variables. Data of P/A ratio, Sim-K, central corneal thickness (CCT), anterior chamber volume (ACV), SE and age were expressed as mean ± standard deviation (SD). Kruskal-Wallis test was used to compare P/A ratio, Sim-K, CCT, ACV, SE, and age among different ophthalmic centers. The correlations between P/A ratio and ΔK, and between P/A ratio or ΔK and other corneal biometrics were analyzed using Spearman's correlation test. Kruskal-Wallis test was used to compare the differences in the distribution of P/A ratio and ΔK in different groups. The level of statistical significance was set at *P* < 0.05.

## Results

A total of 7,893 patients were included (2,340 patients from GZ, 2,255 patients from SY, 1,480 patients from CD, 1,511 patients from WH and 307 patients from HK), consisting of 4,416 males (55.9%) and 3,477 females (44.1%). Mean age of the patients was 25.14 ± 5.41 years. Mean SE of the eyes was −5.13 ± 2.05 D. Age, gender, SE, P/A ratio, Sim-K, CCT, and ACV in the five ophthalmic centers were significantly different (all *P* < 0.0001). Demographic data of the eyes were shown in Table 1.

**Table 1:** Demographics of the patients in the five ophthalmic centers.

**Demographics**	**Ophthalmic centers**						
	**GZ**	**SY**	**WH**	**CD**	**HK**	**Pooled**	*P*-value^*^
Patients^a^	2,340 (29.6%)	2,255 (28.6%)	1,511 (19.1%)	1,480 (18.8%)	307 (3.9%)	7,893 (100.0%)	N/A
Female^a^	1,254 (36.1%)	793 (22.8%)	749 (21.5%)	570 (16.4%)	111 (3.2%)	3,477 (44.1%)	<0.001
Male^a^	1,086 (24.6%)	1,462 (33.1%)	762 (17.3%)	910 (20.6%)	196 (4.4%)	4,416 (55.9%)	<0.001
Age (years)^b^	26.94 ± 5.42	23.88 ± 5.15	25.39 ± 5.03	24.19 ± 5.46	23.97 ± 4.78	25.14 ± 5.41	<0.001
SE (D)^b^	−5.17 ± 2.18	−4.81 ± 1.71	−5.28 ± 1.93	−5.27 ± 2.23	−5.65 ± 2.68	−5.13 ± 2.05	<0.001
P/A ratio^b^	0.81 ± 0.014	0.82 ± 0.014	0.82 ± 0.013	0.81 ± 0.014	0.82 ± 0.012	0.82 ± 0.014	<0.001
Sim-K^b^	42.05 ± 1.32	41.83 ± 1.26	41.86 ± 1.32	41.88 ± 1.31	41.76 ± 1.42	41.91 ± 1.31	<0.001
CCT^b^	542.34 ± 28.62	545.44 ± 28.39	540.59 ± 27.49	545.87 ± 29.14	543.80 ± 29.75	543.61 ± 28.55	<0.001
ACV^b^	195.40 ± 31.50	207.84 ± 31.64	197.26 ± 29.72	203.57 ± 31.74	206.99 ± 30.77	201.29 ± 31.66	<0.001

a *Presented as number (%)*.

b *Presented as mean ± standard deviation*.

* *Comparison among the five ophthalmic centers using Kruskal-Wallis test*.

*SE, Spherical equivalent; D, diopter; P/A ratio, Posterior to anterior corneal radii ratio; Sim-K, Simulated corneal curvature; CCT, Central corneal thickness; ACV, Anterior chamber volume; GZ, Guangzhou aier eye hospital; SY, Shenyang aier eye hospital; WH, Wuhan aier eye hospital; CD, Chengdu aier eye hospital; HK, Hankou aier eye hospital*.

**Figure 1:**
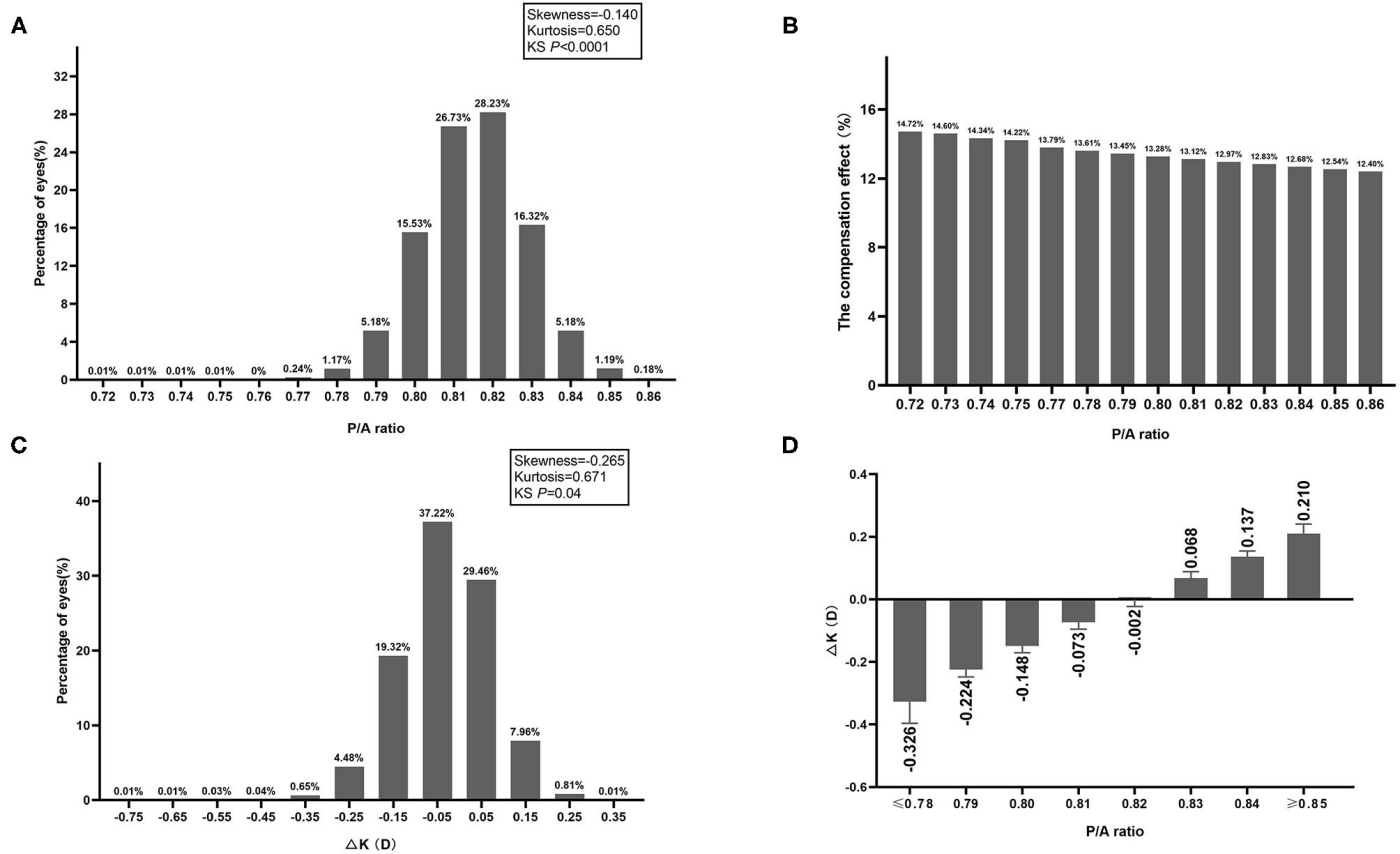
**(A)** Frequency distribution of P/A ratio. **(B)** The compensation effect of posterior refractive power on anterior refractive power in different P/A ratio groups. **(C)** Frequency distribution of difference between True-K and Sim-K (ΔK). **(D)** Difference between True-K and Sim-K (ΔK) in different P/A ratio groups.

P/A ratio distribution was slightly negatively skewed (skewness = −0.140, kurtosis = 0.650, KS *P* < 0.0001).The average P/A ratio was 0.82 ± 0.01 (95% normal range: 0.79–0.84) with a 95% confidence interval (CI) of 0.8148–0.8154 and a coefficient of variance of 1.66%. Among all eyes, 6.6% have values of P/A ratio ≤ 0.79, 86.8% have values of 0.80 ≤ P/A ratio ≤ 0.83, 6.6% have values of P/A ≥ 0.84 (Figure 1A). With the increase of P/A ratio, the compensation effect of posterior refractive power on anterior refractive power was gradually decreased (Figure 1B). ΔK distribution was also slightly negatively skewed (skewness = −0.265, kurtosis = 0.671, KS *P* < 0.0001) (Figure 1C). The mean ΔK ranged from −0.33D to 0.21D, and with greater deviation of the P/A ratio from 0.82, the greater the ΔK deviated from 0 (Figure 1D).

**Figure 2:**
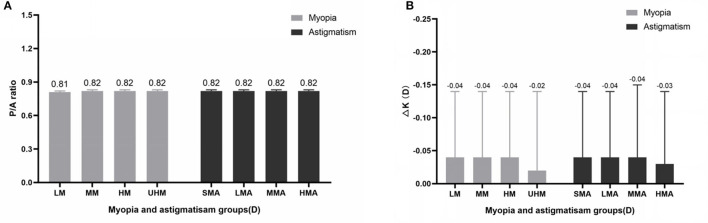
**(A)** P/A ratio in different myopia and astigmatism groups. **(B)** Difference between True-K and Sim-K (ΔK) in different myopia and astigmatism groups.

**Figure 3:**
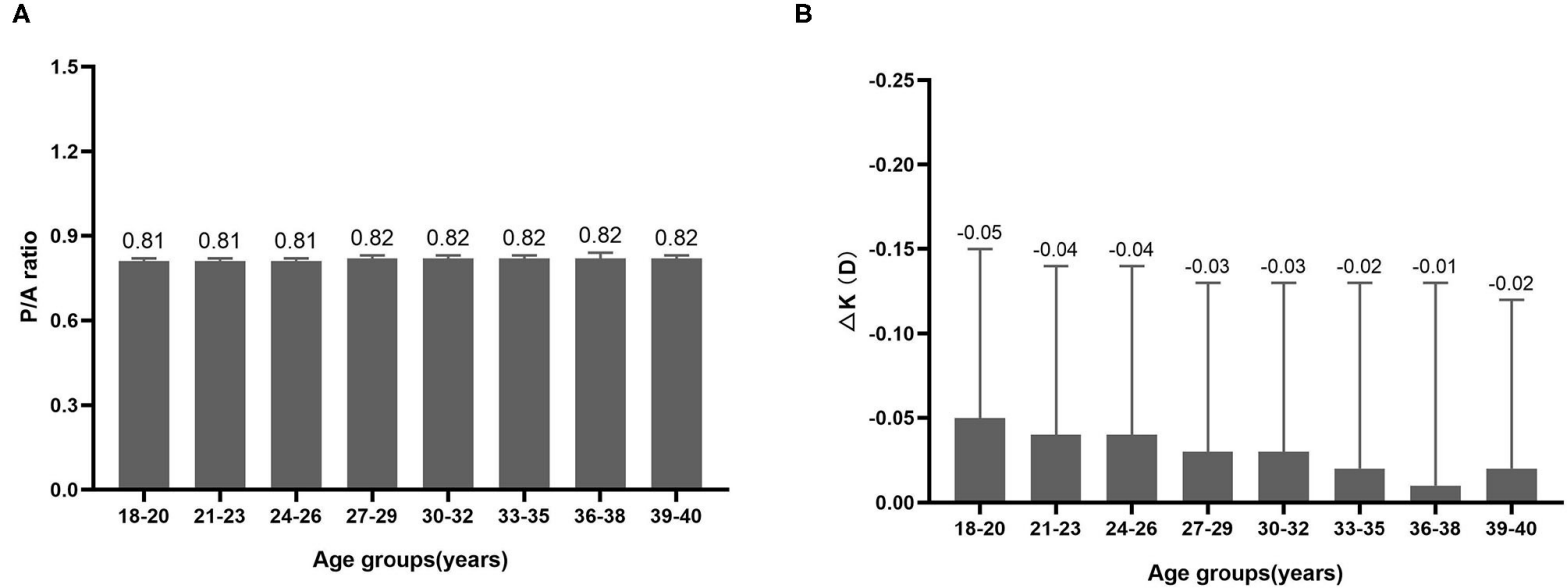
**(A)** P/A ratio in different age groups. **(B)** Difference between True-K and Sim-K (ΔK) in different age groups.

The differences in P/A ratio in different myopia and astigmatism groups were almost negligible (Figure 2A). Although mean ΔK seemed to be lower in the UHM and HMA groups compared with other myopia and astigmatism groups, the difference was also minor (Figure 2B). We further analyzed the P/A ratio and ΔK in different age groups as shown in Figure 3. P/A ratio was similar in different age groups, while ΔK showed a slight trend of decrease with the increase of age.

**Figure 4:**
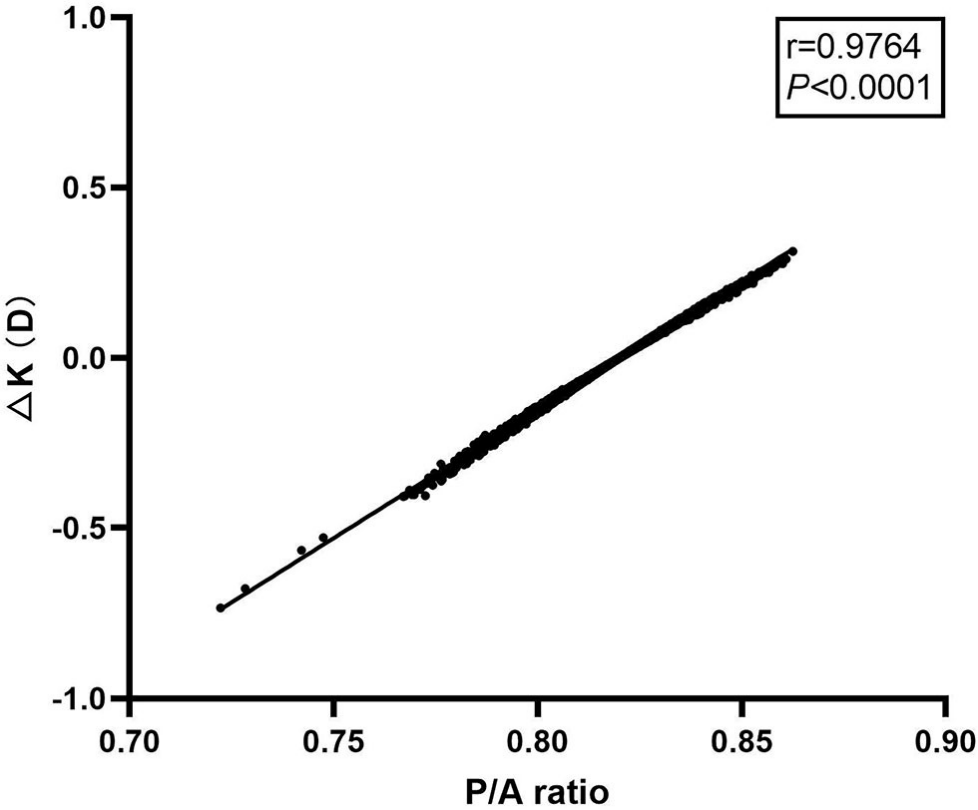
Scattergram showing correlation between posterior to anterior corneal radii ratio and ΔK in all of the eyes.

There was a significant correlation between P/A ratio and ΔK in all of the eyes as shown in Figure 4 (*r* = 0.9764, *P* < 0.0001). A simple linear regression model was applied to analyze the influence of P/A ratio on ΔK. The regression equation is: ΔK = 7.548 × P/A ratio-6.191 (*P* < 0.0001). Thus, a change of 0.1 in P/A ratio would lead to a change of 0.75 D in ΔK.

Correlation coefficients between P/A ratio or ΔK and other corneal biometrics were shown in Table 2. P/A ratio was positively correlated with mean posterior corneal radius (*r* = 0.482), posterior corneal asphericity (*r* = 0.373), corneal diameter (*r* = 0.369), anterior chamber depth (ACD, *r* = 0.215), and ACV (*r* = 0.302). P/A ratio was negatively correlated with posterior corneal eccentricity (*r* = −0.363), CCT (*r* = −0.402), and corneal volume (CV) at 3 mm (*r* = −0.438), 5 mm (*r* = −0.512) and 7 mm (*r* = −0.594) areas. ΔK was positively correlated with mean posterior corneal radius (*r* = 0.505), posterior corneal asphericity (*r* = 0.380), ACD (*r* = 0.314), and ACV (*r* = 0.383). ΔK was negatively correlated with posterior corneal eccentricity (*r* = −0.370), CCT (*r* = −0.410), and CV at 3 mm (*r* = −0.447), 5 mm (*r* = −0.524) and 7 mm (*r* = −0.609) areas.

**Table 2:** Summary of Spearman's correlation analysis between P/A ratio or ΔK and other corneal biometrics.

**Parameters**	**P/A ratio**			**ΔK**		
	***r***	**95% CI**	***P***	***r***	**95% CI**	***P***
Spherical equivalent	−0.014	(−0.036, 0.009)	0.230	−0.014	(−0.036, 0.009)	0.227
Mean anterior corneal radius	0.061	(0.039, 0.084)	<0.001	0.077	(0.054, 0.100)	<0.001
Anterior corneal astigmatism	−0.069	(−0.091, −0.046)	<0.001	−0.077	(−0.100, −0.055)	<0.001
Anterior corneal eccentricity	0.001	(−0.022, 0.024)	0.938	−0.004	(−0.027, 0.019)	0.729
Anterior corneal asphericity	0.003	(−0.020, 0.026)	0.791	0.008	(−0.014,0.031)	0.462
Mean posterior corneal radius	0.482	(0.465, 0.500)	<0.001	0.505	(0.488, 0.522)	<0.001
Posterior corneal astigmatism	−0.215	(−0.236, −0.193)	<0.001	−0.224	(−0.246, −0.203)	<0.001
Posterior corneal eccentricity	−0.363	(−0.383, −0.343)	<0.001	−0.370	(−0.389, −0.350)	<0.001
Posterior corneal asphericity	0.373	(0.354, 0.393)	<0.001	0.380	(0.361, 0.400)	<0.001
Central corneal thickness	−0.402	(−0.421, −0.383)	<0.001	−0.410	(−0.429, −0.391)	<0.001
Corneal volume−3 mm	−0.438	(−0.456, −0.419)	<0.001	−0.447	(−0.465, −0.429)	<0.001
Corneal volume−5 mm	−0.512	(−0.529, −0.495)	<0.001	−0.524	(−0.540, −0.507)	<0.001
Corneal volume−7 mm	−0.594	(−0.609, −0.579)	<0.001	−0.609	(−0.623, −0.594)	<0.001
Corneal diameter	0.369	(0.349, 0.388)	<0.001	0.219	(0.198, 0.241)	<0.001
Anterior chamber depth	0.215	(0.193, 0.236)	<0.001	0.314	(0.293, 0.334)	<0.001
Anterior chamber volume	0.302	(0.281, 0.323)	<0.001	0.383	(0.364, 0.402)	<0.001

*P/A ratio, Posterior to anterior corneal radii ratio; ΔK, Difference between True-K and Sim-K; CI, Confidence interval*.

## Discussion

In the present study, we demonstrated the distribution pattern of P/A ratio in Chinese myopia patients from multiple ophthalmic centers. Mean P/A ratio in our study was 0.82 ± 0.01 with a range of 0.72–0.86. The results were consistent with previous studies where the range of P/A ratio was 0.81–0.84 in the normal human eye (23–25). The wider range of P/A ratio in our study may be due to the relatively larger sample size participants from multiple centers.

Our study clearly revealed that 6.6% of eyes have values of P/A ratio ≤ 0.79 and 6.6% of eyes have values of P/A ≥ 0.84, resulting in a discrepancy of ≥0.25 D between Sim-K and True-K in 5.29% of eyes, and the ΔK could range from −0.73 to 0.31 D. The ΔK was also found to be correlated with the P/A ratio and a change of 0.1 in P/A ratio could lead to a change of 0.75 D in ΔK. Such effects indicated that it would be important to measure the anterior and posterior curvature radii of the cornea to obtained the true P/A ratio. Sim-K or other CRP formulas neglecting the actual power of the posterior cornea may be not enough to accurately calculate the CRP. Mingue Kim et al. investigated 158 cataract patients and found that the postoperative refractive prediction error within ± 0.50 D improved significantly from 62.7 to 74.7% when using the actual P/A ratio and applying the True-K instead of Sim-K to the Haigis formula to calculate the IOL power. Thus, using CRP formulas based on the actual P/A ratio provides more accurate postoperative refraction than that using Sim-K (12).

It has been suggested that the clinical relevance of using P/A adjusted corneal power may be limited in normal eyes (12), and it is also possible that the measurement error of Pentacam examination may weight out the benefits of P/A adjusted corneal power in normal eyes (21). But for patients with large P/A deviation such as those with previous keratoplasty, keratoconus, or corneal refractive surgery, the P/A adjusted corneal power would be beneficial (12). For such patients with unusual corneas, the IOL power calculation from Sim-K may be imprecise, causing a hyperopic postoperative refractive error, while the True-K that considering both the anterior and posterior corneal curvatures is more accurate for IOL power calculation in these cases. As demonstrated by Tamaoki et al., the corneal power obtained from actual P/A ratio and corneal thickness was applied to the IOL power formula SRK/T in eyes with posterior keratoconus and yielded a better postoperative refractive outcome (15). Results of our study may also provide evidence about the normal range of P/A ratio when deciding what is a deviated P/A value.

CRP calculation is also important in eyes with previous corneal refractive surgery. The P/A ratio is significantly changed after corneal refractive surgery (e.g., SMILE, LASIK, PRK) in which central corneal tissue is ablated to flatten the anterior corneal surface, resulting in the deviation of P/A ratio from 0.82 (26–28). In these special cases, IOL power is usually underestimated if calculated based on the Sim-K (29, 30). Corneal refractive surgery reduces CCT and anterior corneal curvature, thereby lowering the P/A ratio, which renders overestimation of the CRP by Sim-K, leading to a biased IOL power calculation and as a consequence the patients having a risk of postoperative hyperopia (16). Schuster et al. showed that in eyes after laser refractive surgery, the error of IOL power calculation was related to the P/A ratio. Using regression analysis the authors showed that P/A ratio was a significant influencing factor associated with the error of IOL power calculation (the Holladay 1 formula reg β = −8.69, SRK/T formula regβ = −10.81) (31). Previous studies have demonstrated that True-K is significantly different from Sim-K in eyes with previous corneal refractive surgery, and True-K is more accurate for IOL power calculation compare to Sim-K (32, 33). In the present study we found that the difference between Sim-K and True-K was not a constant, but depended on the P/A ratio. Our findings were consistent with a previous study which reported that ΔK was strongly correlated with P/A ratio, and had an impact on the refractive outcome of IOL power calculation (34). Therefore, our results shed light on the importance of determining IOL power using the actual P/A ratio in patients with previous corneal refractive surgery.

P/A ratio and corneal wavefront aberrations are also changed in eyes after keratoplasties (35–37). In normal eyes, the anterior and posterior surfaces of the cornea are parallel, and the posterior surface compensates the high-order aberrations (HOAs) of the anterior surface. However, it's suggested that the normal parallelism breaks down, resulting in degraded modulation transfer functions and increased corneal HOAs including spherical aberration, leading to decreased visual acuity compared to normal subjects (35, 36). Theoretically, change of P/A ratio after keratoplasties can influence spherical aberration leading to refractive outcomes. It has also been shown that P/A had the highest correlation with the change in corneal refractive power and could be used to identify eyes that might be at risk of a greater postoperative hyperopic shift after Descemet membrane endothelial keratoplasty (37). However, which components of the HOA are affected by P/A ratio change still need to be further investigated.

In our study, we found that P/A ratio and ΔK were positively correlated with the mean posterior corneal radius, and the correlation coefficients were 0.482 and 0.505, respectively. Since P/A ratio is defined as the ratio of posterior to anterior curvature radii of the cornea, ΔK is the difference between True-K and Sim-K. Therefore, P/A ratio and ΔK increase as the radius of the posterior corneal surface increases. The P/A ratio and ΔK were negatively correlated with CCT and CV. This means the thinner the cornea, the greater the P/A ratio. The finding is consistent with the results of a previous study which also revealed a similar trend (24). In addition, our research found that P/A ratio and ΔK were positively correlated with ACD, ACV, and posterior corneal asphericity, while negatively correlated with posterior corneal eccentricity. It is possible that shape of the posterior cornea is affected by a higher ACD and ACV, causing a higher P/A ratio. Or it could be the other way around, an elevated posterior corneal surface causes more posterior corneal eccentricity and deepening of the anterior chamber. Researchers have found that ACD is significantly deeper in eyes with keratoconus, a disease that is characterized by continuous protrusion of the posterior cornea at the early stage (38).

A recent study showed that ambient environment can have influence on the CCT and ACV in young adults. People who lived at high latitudes for a long time had thinner CCTs and greater ACVs. And individuals who grew up in warm and wet environments had thicker CCTs and smaller ACV (39). It seemed that CCT and ACV had different directions of change in response to ambient environment alteration. Since the anterior cornea is directly exposed to the ambient environment and its change may be similar with CCT. But the posterior cornea is in direct contact with the aqueous humor and its change may be similar with ACV. Theoretically, environmental factors may also have an impact on the P/A ratio.

A previous study showed that the P/A ratio was negatively correlated with age, i.e., elder participants had lower P/A ratio (40). But a positive correlation was found between the P/A ratio and age in another study (41). With the growth of age, the anterior corneal surface is steepened in the center and flattened toward the periphery, while the posterior corneal radius remains unchanged (23, 42), therefore the P/A ratio of the central cornea seems to be increased with aging. However, in the present study the P/A ratio was not correlated with age. This might be because of a narrow age range in our study (18–40 years).

There are some limitations of our study. Firstly, this study can be biased due to selection of subjects in the candidates for myopic refractive surgery, and our conclusion needs to be validated in older subjects, who are the candidates of cataract surgery. Secondly, this study is retrospective. Future prospective studies are needed to investigate the true impact of P/A ratio on the outcomes of refractive and cataract surgery.

In conclusion, P/A ratio follows a specific distribution pattern in myopic Chinese patients, rather than being a constant of 0.82. The larger the actual P/A ratio is deviated from 0.82, the larger the difference is between True-K and Sim-K (ΔK). In myopic refractive and cataract surgery, it is important to emphasize measurement of the posterior corneal radius of curvature.

## Data Availability Statement

The data analyzed in this study is subject to the following licenses/restrictions: The raw data supporting the conclusions of this article will be made available by the corresponding authors on reasonable request. Requests to access these datasets should be directed to huyijun2014@163.com.

## Ethics Statement

This retrospective study conformed to the tenets of the Declaration of Helsinki and was approved by the Institutional Review Board (IRB) of Guangzhou Aier Eye Hospital (GZ), Shenyang Aier Eye Hospital (SY), Wuhan Aier Eye Hospital (WH), Chengdu Aier Eye Hospital (CD), and Hankou Aier Eye Hospital (HK). It was only a review of medical records and patients could not be identified from the data, so the IRBs decided to waive the need to obtain informed consent.

## Author Contributions

YH, HY, and CT designed this study and provided intellectual content of critical importance to the work described. CT, QW, BL, GW, and JF analyzed and interpreted the data and discussed the results and commented on the manuscript. CT wrote the article. YH and HY revised the manuscript. All authors approved the final manuscript to be published.

## Funding

This work was supported by Grant 81870663 from the National Natural Science Foundation of China (HY), Grant KJ012019087 of the Outstanding Young Talent Trainee Program of Guangdong Provincial People's Hospital (HY), Grant KJ012019457 from the GDPH Scientific Research Funds for Leading Medical Talents and Distinguished Young Scholars in Guangdong Province (HY), Grant Y012018145 from the Talent Introduction Fund of Guangdong Provincial People's Hospital (HY), Grant A2021378 from the Medical Scientific Research Foundation of Guangdong Province, China (YH), Grant 2018SK50106 from the Technology Innovation Guidance Program of Hunan Province (YH), and Grant AM1909D2 and AR1909D2 from the Science Research Foundation of Aier Eye Hospital Group (YH).

## Conflict of Interest

The authors declare that the research was conducted in the absence of any commercial or financial relationships that could be construed as a potential conflict of interest.

## Publisher's Note

All claims expressed in this article are solely those of the authors and do not necessarily represent those of their affiliated organizations, or those of the publisher, the editors and the reviewers. Any product that may be evaluated in this article, or claim that may be made by its manufacturer, is not guaranteed or endorsed by the publisher.
